# Preventing malaria in international travellers: an evaluation of published English-language guidelines

**DOI:** 10.1186/1471-2458-14-1129

**Published:** 2014-11-03

**Authors:** Merav Kliner, Kristina Poole, David Sinclair, Paul Garner

**Affiliations:** Cheshire and Merseyside Public Health England Centre, 5th Floor, Rail House, Lord Nelson Street, Liverpool, L1 1JF UK; Liverpool School of Tropical Medicine, Pembroke Place, Liverpool, L3 5QA UK

**Keywords:** Malaria, Travellers, Chemoprophylaxis, Prevention

## Abstract

**Background:**

People intending to travel may seek information on malaria prevention from a range of sources. To ensure the best protection, this information needs to be reliable, up-to-date, consistent, and useful to their decision making. This study appraises current international and national guidelines written in English for malaria prevention in travellers, and whether any recommendations conflict.

**Methods:**

We systematically identified national or international English-language guidelines on malaria prevention in travellers to July 2013 using standard and multiple searching methods. We critically appraised guidelines using the AGREE II tool, and report inconsistent recommendations within guidelines.

**Results:**

We identified five sets of English-language guidelines on preventing malaria for travellers. Assessment against AGREE II indicate that all of the guidelines fall short of internationally accepted standards in guideline development: none include a transparent description of methods; only one describes sources of funding or potential conflicts of interest; and only one includes formal presentation of the evidence alongside transparent assessment of the quality of that evidence. There were a number of important discrepancies between guidelines, and some omit information about effectiveness, safety and adverse effects of chemoprophylaxis options.

**Conclusions:**

The methods used for developing guidelines for malaria prevention in travellers lags behind current internationally recognized standards. Healthcare professionals as well as travellers themselves could be better informed if guidelines were more systematic and transparent summaries of the current knowledge on drug interventions in relation to effects, safety, administration and contra-indications.

**Electronic supplementary material:**

The online version of this article (doi:10.1186/1471-2458-14-1129) contains supplementary material, which is available to authorized users.

## Background

In the UK, approximately 2,000 episodes of malaria are recorded each year in people returning from travel to endemic countries, and travellers still die from malaria [1]. Travellers can reduce their risk of malaria illness by taking antimalarial drugs throughout their time abroad as prophylaxis, and by various non-drug interventions which reduce mosquito bites.

People intending to travel may access information on malaria prevention through on-line resources, by visiting their own doctor, through specialist travel clinics, or through various travel operators [2]. To ensure the best protection, this information needs to be reliable, up-to-date and based on the best available research evidence. In the UK, health workers will usually follow guidelines developed by Public Health England, or available through on-line travel sites such as the National Travel Health Network and Centre (NaTHNaC). However, many other guidelines are available developed by other national or international bodies.

Travellers to malaria endemic settings require information that enables them, and convinces them, to choose the interventions that are most effective and safe for them. This is likely to involve some quantification of the relative benefits and harms of each intervention. To facilitate this, good quality guidelines should present this information in a clear and user-friendly format [3].

In this study, we aim to a) appraise the available national and international English-language guidelines on malaria prevention against international best practice in guideline development, b) evaluate the degree of consistency or conflict between guidelines, and c) consider how well the information facilitates informed decision making.

## Methods

### Identification of guidelines

Two authors (MK, KP) searched independently for international or national guidelines for malaria prevention in travellers using: Google, the websites of major public health bodies from English-speaking non-endemic countries (the UK, USA, Australia, New Zealand, Scotland and Canada), the TRIP database, the National Guideline Clearing House, and pubmed (using the search terms: malaria AND (prevent* OR prophylax*) AND guid* AND travel) for articles published until the end of July 2013. Guidelines limited to malaria prevention in sub-groups (such as children), or in languages other than English, were excluded.

### Agree II appraisal of guidelines

Two authors (MK, KP) independently appraised each identified guideline using the AGREE II guideline appraisal tool [4]. The AGREEII tool appraises guidelines against 23 criteria across six domains; scope and purpose, stakeholder involvement, rigour of development, clarity of presentation, applicability, and editorial independence. Each reviewer first assigned a score between one and seven for each criteria (one being the lowest and seven the highest), and the scores of both reviewers were then combined and converted into a percentage score for each domain.

### Agreement between guidelines

Two authors (MK, KP) independently reviewed each guideline to identify both explicit and implicit recommendations relating to prevention of malaria. Explicit recommendations were classified as those where something was clearly being advised (for example, “Spraying before bedtime, combined with the use of a vaporiser is recommended”). Implicit recommendations were classified as statements where judgements are being made (for example, “Insect repellent applied to clothing is effective for longer than it may be on the skin”). Two reviewers mapped recommendations to a standardised table independently and compared and contrasted them. Discrepancies were addressed through discussion between the two reviewers and where discrepancies could not be resolved, a third reviewer (DS) was consulted. Two reviewers (MK, KP) compared the recommendations made by each of the guidelines to identify any conflicts between sets of guidelines.

## Results

Five guidelines met our inclusion criteria, developed by: the World Health Organization (WHO) [5], the Public Health Agency of Canada (CA) [6], the Hong Kong Centre for Health Protection (HK) [7], the Public Health England advisory committee on malaria prevention (UK) [8] and the Centre for Disease Control (USA) [9].

We excluded an article providing advice for travellers from New Zealand, as it was not official national guidance [10], and although our PUBMED search suggested there may be guidelines from Japan, The Netherlands and Spain, these were unavailable in English. No guidance was identified from Australia or Scotland. A brief summary of these guidelines is outlined in Table 1.

**Table 1:** **Brief summary of guidelines**

	Canada	Hong Kong	UK	USA	WHO
**Year**	2009	2007	2013	2013	2012
**Format**	Stand-alone guidance	Stand-alone guidance, two documents	Stand-alone guidance	Within ‘Yellow Book’ on travel health, chapter on malaria (chapter 3) and prevention against mosquitos (chapter 2)	Within ‘International travel and health’; chapter on ‘malaria’ and subsection on ‘Insects and other vectors of disease’
**Development team**	Committee to Advise on Tropical Medicine and Travel (7 named individuals)	Working Group on Malaria Prophylaxis (5 named authors, 2 collaborating groups)	Public Health England Advisory Committee on Malaria Prevention in UK travellers (5 named authors, 6 named contributors	6 named chapter authors, with wide range of editorial staff, CDC and external contributors	2 editors, 3 assistants and a wide range of WHO and external personnel
**Scope**	Drug and non-drug prevention of malaria in travellers	Drug and non-drug prevention of malaria in travellers	Drug and non-drug prevention of malaria in travellers	Drug and non-drug prevention of malaria in travellers	Drug and non-drug prevention of malaria in travellers
**Length**	90 pages	Split into two papers, total 58 pages	97 pages	Split into 2 chapters, total 22 pages	Split into 2 chapters, total 26 pages

### Agree II appraisal of guidelines

The summary AGREE II scores for each of the five guidelines are shown in Table 2.

**Table 2:** **AGREE II scores for guidelines**

AGREE II domain	Canada	Hong Kong	UK	USA	WHO
Domain 1: Scope and purpose^1^	14%	72%	92%	44%	50%
Domain 2: Stakeholder involvement^2^	17%	33%	58%	39%	53%
Domain 3: Rigour of development^3^	20%	9%	14%	22%	12%
Domain 4: Clarity of presentation^4^	72%	61%	53%	47%	50%
Domain 5: Applicability^5^	4%	4%	13%	13%	4%
Domain 6: Editorial independence^6^	0%	0%	0%	50%	0%

^1^Scope and purpose concerns the overall aim of the guideline, the scope of the questions, and the target audience.

^2^Stakeholder involvement looks at the extent to which the guideline development process included the views of all appropriate stakeholders, including the intended users of the guideline and those affected by the recommendations.

^3^Rigour of development examines the process used to search for, synthesize, and appraise evidence, formulate recommendations, and keep them updated.

^4^Clarity of presentation concerns the general language, structure, and format of the guideline.

^5^Applicability requires adequate consideration of the likely barriers and facilitators to implementation, including resource considerations, and advice or tools to improve uptake and implementation.

^6^Editorial independence concerns the adequate declaration and management of potential conflicts of interest related to the funding body or the guideline group members.

#### Domain 1: Scope and purpose

All five guidelines covered both drug and non-drug interventions to prevent malaria in travellers, and all aimed to assist clinicians as they advise travellers. The UK and HK guidelines provided the most complete description, clearly outlining the objectives, health issue and target population to which the guidelines apply.

#### Domain 2: Stakeholder involvement

All guidelines gave a named list of the individuals who primarily authored the document, although only the UK guidelines stated the roles and responsibilities of these individuals. Three guidelines (HK, USA and WHO) also referred to working groups or external contributors but these were not named. None of the guidelines reported involvement of the target population (travellers).

#### Domain 3: Rigour of development

None of the guidelines included a Methods section. None described methods to systematically search for and appraise evidence, or to move from evidence to formulating recommendations. None of the documents described external peer review prior to publication, and only the WHO guideline made reference to a process for future updates.

The Canadian guideline presented the evidence base summarized in GRADE tables. All other guidelines summarized evidence in text form, with variable levels of referencing provided to validate recommendations. USA and WHO guidelines provide no references, HK provide some references and UK guidelines provide a reference for many of the recommendations made.

All guidelines discussed the health benefits and side effects of interventions to some extent, although the level of detail, particularly around safety was highly variable. The Canadian and WHO guidelines gave some risk estimates for the potential benefits and harms.

#### Domain 4: Clarity of presentation

All of the guidelines presented some clear recommendations that were unambiguous and specific, and clearly identifiable. However, all guidelines also included discussions of options without clear recommendations, or implied recommendations incorporated into the text and without clear signposting. The Canadian guidelines were presented most clearly, listing all explicit recommendations in a table.

#### Domain 5: Applicability

None of the guidelines discussed the resource implications of the recommendations, and none described potential barriers or facilitators to implementation. None presented audit criteria. Only the USA guideline presented an outline of the advantages and disadvantages of each drug in a user-friendly table.

#### Domain 6: Editorial independence

Only the USA guideline described the sources of funding, and made any reference to potential conflicts of interest among guideline developers. The remaining guidelines said nothing, and therefore scored zero for this domain.

### Agreement or disagreement between guidelines

#### a. Drug interventions for malaria prevention

A summary of the drug recommendations made by each of the guidelines has been presented in Additional file 1. The main areas of conflict and omission are related to information on effectiveness, safety and contraindications:

- All guidelines state that atovaquone-proguanil, doxycycline and mefloquine are effective against chloroquine resistant falciparum malaria. Two guidelines quantify this: the Canadian guidelines state that they are 95% effective and the UK guidelines state that they are 90% effective, each referencing different documents.
- The Canadian, Hong Kong and USA guidelines state that atovaquone-proguanil has an excellent safety profile, with only the Canadian guidelines quantifying that 8% to 15% of individuals experience nausea, vomiting, abdominal pain or diarrhoea. The guidelines state that the most common side effects are headache and gastrointestinal upsets, but again do not quantify this.
- Although the Canadian guideline outlines that atovaquone-proguanil has an excellent safety profile, it outlines that it should be used in caution in patients with HIV and liver disease. This is not discussed in other guidelines;
- The US guideline states that atovaquone-proguanil cannot be used in children under 5 kg, whereas all other guidelines state 11 kg as the cut-off;
- All guidelines state that doxycycline may be photosensitising and can lead to a rash, however no guidelines quantify this risk;
- All guidelines state that doxycycline may lead to gastrointestinal upset and oesophageal ulceration, however no guidelines quantify this risk;
- The UK guideline states that doxycycline is contraindicated in children under 12 years, while all other guidelines say 8 years;
- All guidelines state that mefloquine can lead to major neuropsychiatric side effects such as seizure, psychosis and suicidal ideation. The Canadian and WHO guidelines quantify this. Canadian guidelines suggest that 1 in 6,000 to 13,000 have seizure or psychosis; and WHO guidelines suggest severe neuropsychiatric disturbance in 1 in 10,000.
- The Canadian, Hong Kong and USA guidelines state that mefloquine can lead to minor side effects such as nausea, strange vivid dreams, dizziness, mood changes, insomnia, headache and diarrhoea. The UK and WHO guidelines do not mention this. Only the Canadian guideline quantifies this, suggesting that 95% of people have no or mild, temporary side effects, with 1 in 250 to 500 having mild neuropsychological reactions e.g. anxiety or mood change;
- The US guideline states that mefloquine can be given to all ages, while other guidelines state it cannot be used in children below 5 kg;
- Stand-by treatments is recommended as being valuable in all guidelines, although the USA guidelines provide very little information regarding this.

#### b. Non–drug interventions for malaria prevention

A full outline of the non-drug recommendations made by each of the guidelines has been presented in Additional file 2. There is varying levels of consensus between the guidelines, with a number of significant conflicts.

The main conflicts and omissions are that:

- The WHO guidelines states insecticide-treated clothing lasts longer than skin applied insecticide, whereas UK guidelines states the opposite. Other guidelines provide no recommendations regarding this;
- Concentrations of DEET recommended in children vary from 10% (CA, HK) to 50% (UK), and in adults vary from 35% (CA, HK) to 50% (UK, USA) in adults. WHO guidelines state that DEET should be used according to manufacturers’ recommendations;
- The Canadian and WHO guidelines state that DEET can be used at any age, while the UK and USA guidelines state it should be restricted to children over the age of 2 months. Hong Kong guidelines do not provide recommendations on age limits of DEET;
- The WHO guidelines state that no other protection is required if a hotel is air-conditioned. The UK and USA guidelines imply that malaria transmission is reduced in air-conditioned rooms, and the Canadian and Hong Kong guideline do not provide recommendations on air-conditioning;
- Light covering clothing is recommended by Canada and Hong Kong, while the UK guidelines state this is not effective. USA and WHO guidelines do not provide recommendations on the use of light covered clothing.

## Discussion

Appraisal of the five guidelines using the AGREE II tool indicates that all of the English guidelines surveyed (from national bodies and international bodies) fall short of internationally accepted standards in guideline development. None include a transparent description of methods used, only one describes sources of funding and potential conflicts of interest, and only one includes formal presentation of the evidence alongside transparent assessments of the quality of that evidence. There were a number of important discrepancies between guidelines; and some omit information about effectiveness, safety and adverse effects of chemoprophylaxis options.

Moving from research evidence to healthcare recommendations is not straightforward. High quality evidence that an intervention is effective does not automatically lead to a strong recommendation to implement it, and low quality evidence does not preclude a positive recommendation [11]. The process instead involves a series of judgements by the guideline panel, and may take into account the importance of the condition, the applicability of the evidence to the population group, and cost. These judgements are influenced by the values and preferences of the guideline developers, and consequently it is not unexpected that different guidelines developed by different teams will have discrepancies and inconsistencies as highlighted in this paper. More problematic however, is the fact that in most of these guidelines it is unclear what evidence was reviewed, what the strengths and weaknesses of that evidence was, or the basis of the judgements may be the panels.

A second clear deficiency common across guidelines was the lack of user-friendly resources for implementation. Although most guidelines stated that they should be used by healthcare workers and travellers to identify the most suitable interventions for individuals, most did not provide adequate information to help these decisions between different strategies, and quantified estimates of the proportion of people who would be likely to benefit or be harmed by an intervention were rarely given. This was particularly true of drug interventions where side effects are often one of the most important factor influencing choice [12, 13]. Therefore, they did not give adequate information about when chemoprophylaxis should be used, and if so, which drug should be given. Shared decision making between healthcare workers and patients may be beneficial where evidence is scarce or conflicting and can be encouraged by adapting guidelines to including information for healthcare workers on how to discuss options with patients, and ensuring that the guidelines are accessible to patients such as through patient support tools [14, 15].

In addition there were a number of inconsistencies or discrepancies between guidelines. Most were omissions of information, but some reflected different or conflicting recommendations. Identifying these may suggest this is where the evidence is weak or limited, and expert judgment alone has been the main driver of the recommendation. Being clear and transparent about the presence or absence of high quality evidence behind a recommendation helps identify priority research questions, and stimulate a genuinely useful research agenda.

One limitation of this study is that we only included English-language guidelines. Italian guidelines, published as a summary in English after the literature search was conducted for this paper, outlines that national and internationals guidelines have moved in a varying degree away from routinely recommending chemoprophylaxis to a greater use of a wide range of preventative methods [16]. There may be a difference in recommendations between English-language and non-English-language guidelines and using these methods in a cross-language comparison may provide help in assuring guideline quality and standardising recommendations for preventing malaria.

## Conclusions

We evaluated five sets of guidelines written in English on the prevention of malaria for travellers. Guidelines had a number of omissions and conflicts, and were not clearly based on evidence. Healthcare professionals and travellers would benefit from consistent, easy to use guidelines, based clearly on evidence, and outlining current knowledge on effectiveness of interventions, safety, administration and contra-indications.

## Electronic supplementary material

Additional file 1: **Consensus between guidelines for drug interventions.** (DOC 144 KB)

Additional file 2: **Consensus between guidelines for non-drug interventions.** (DOC 115 KB)

## Abbreviations

CA: Canada
HK: Hong Kong
WHO: World Health Organisation.
