# Supplementary material for: Preventing malaria in international travellers: an evaluation of published English-language guidelines
Source: BMC Public Health. 2014 Nov 3;14:1129. doi: 10.1186/1471-2458-14-1129 (PMC4226897; doi:10.1186/1471-2458-14-1129)
Supplement: Supplementary file 1 — Additional file 1: Consensus between guidelines for drug interventions. (DOC 144 KB) [file 12889_2014_7210_MOESM1_ESM.doc]

***Additional file 1:*** ***Consensus between guidelines for drug interventions***

| Drug Interventions | Advice | Does guideline make recommendation? | | | | |
| --- | --- | --- | --- | --- | --- | --- |
|  |  | CA | HK | UK | USA | WHO |
| CHLOROQUINE | | | | | | |
| Effectiveness | Use in chloroquine-sensitive areas | Y | Y | Y | Y | Y |
|  | Use for most P. *vivax*, all P. *ovale*, P*. knowlesi*, and most P*. malariae* | - | - | Y | - | Y^a^ |
| Administration | Take once weekly | Y | - | Y | Y | Y |
|  | Take 1 week before entering malarial region, during period of exposure, and for 4 weeks after leaving malarial region | Y | Y | Y | Y | Y |
| Safety | Chloroquine is suitable for all ages | Y | Y | Y | Y | Y |
|  | Chloroquine is suitable for pregnant women | Y | Y | Y | Y | Y |
|  | 25-50% will experience side effects from chloroquine | Y | - | - | - | - |
|  | Minor side effects include nausea and headache | - | Y | Y | Y | - |
|  | May initially cause transient, minor visual blurring | Y | - | - | Y | - |
|  | Retinal toxic effects are extremely unlikely | Y | Y | Y | Y | Y |
|  | Rarely associated with seizures and psychosis | Y | - | - | - | - |
|  | Caution for patients with psoriasis, as it may worsen condition | Y | - | Y | Y | - |
|  | Caution for patients with myasthenia gravis, as it may worsen condition | - | - | Y | - | - |
|  | Caution with black-skinned people, as it may cause generalized pruritus | Y | - | Y | - | - |
|  | Caution with amiodarone, ciclosporin, digoxin, mefloquine, moxifloxacin, zyban | - | - | Y | - | - |
| Contra-indications | Contraindicated in those with epilepsy or generalized psoriasis | Y | Y | Y | - | Y |
|  | Contraindicated in those with G6PD deficiency | - | - | Y | - | - |
|  | Do not give with live vaccines (in particular intra-dermal rabies vaccine) within 8 hours of chloroquine | Y | - | Y | - | Y |
| Chloroquine-proguanil | | | | | | |
| Effectiveness | Use in areas with P*. vivax* and *P. falciparum* malaria, with emerging chloroquine resistance | - | - | N^b^ | - | Y |
| Administration | Take chloroquine once weekly and proguanil once daily | - | - | - | - | - |
|  | Take 1 week before entering malarial region, during period of exposure, and for 4 weeks after leaving malarial region | - | Y | Y | - | Y^c^ |
| Safety | Can be used in pregnancy and children | Y | Y | Y | - | Y |
|  | May cause oral aphthous ulcerations | Y | Y | Y | - | - |
| Contra-indications | Do not give with live vaccines | Y | - | - | - | Y |
| Atovaquone-proguanil | | | | | | |
| Effectiveness | Use atovaquone-proguanil in areas with chloroquine-resistant malaria | Y | Y | - | Y | Y |
|  | Use atovaquone-proguanil in areas with mefloquine-resistant malaria | Y | - | - | Y | - |
|  | Atovaquone-proguanil is 90% or more effective against malaria | Y | - | Y | - | - |
| Administration | Take once daily | Y | - | Y | Y | Y |
|  | Take 1 day before entering malarial region, during period of exposure, and for 1 week after leaving malarial region | Y | Y | Y | Y | Y |
|  | Take with food or milky drink to increase absorption | - | - | - | - | Y |

| Safety | Excellent safety and tolerance profile | Y | Y^d^ | - | Y | - |
| --- | --- | --- | --- | --- | --- | --- |
|  | May cause nausea, vomiting, abdominal pain or diarrhoea | Y^e^ | Y | Y | Y | - |
|  | May develop elevations in transaminase and amylase levels | Y | - | - | - | - |
|  | May rarely cause seizure, hepatitis and rash | Y | - | - | - | - |
|  | Caution in HIV infected patients as may cause rash and fever | Y | - | - | - | - |
|  | Caution with anticoagulants, as may potentiate the effect | Y | - | Y | Y | - |
| Contra-indications | Contraindicated in severe renal insufficiency | Y | - | Y | Y | Y |
|  | Do not give with live vaccines | Y | - | - | - | Y |
|  | Contraindicated with efavirenz, indinavir, zidovudine, rifabutin, rifampicin, tetracycline and metochlopramide | - | - | Y | - | Y |
|  | Contraindicated during pregnancy | Y | Y | Y | Y | Y |
|  | May be given after first trimester in women who cannot avoid travel to mefloquine-resistant areas | Y | - | Y | - | - |
|  | Contraindicated in children less than 11kg | Y | Y | Y | Y^f^ | Y |
|  | Contraindicated in breastfeeding women | Y | - | Y | - | - |
| DOXYCYCLINE | | | | | | |
| Effectiveness | Use doxycycline in areas with chloroquine-resistant malaria | Y | Y | - | Y | Y |
|  | Use doxycycline in areas with mefloquine-resistant malaria | Y | Y | - | Y | - |
|  | Doxycycline is 90% or more effective against malaria | Y | - | Y | - | - |
| Administration | Take once daily | Y | - | Y | Y | Y |
|  | Take 1 day before entering malarial region, during period of exposure, and for 4 weeks after leaving malarial region | Y | Y | Y | Y | Y |
|  | Take with water or food to prevent oesophageal irritation | - | Y | Y | Y | Y |
|  | Use monohydrate rather than hyclate form of the drug, as is better tolerated | - | - | - | - | Y |
| Safety | Caution in sun as doxycycline is photosensitizing, and it may make the skin burn more easily | Y | Y | Y | Y | Y |
|  | May cause vaginal candidiasis | Y | Y | Y | Y | Y |
|  | May cause gastrointestinal upset and oesophageal ulceration | Y | Y | Y | Y | Y |
|  | Caution with anticoagulants, as may potentiate the effect | Y | - | Y | - | - |
|  | Caution with barbiturates, carbamazapine or phenytoin | Y | - | Y | - | - |
|  | Caution in patients with myasthenia gravis and systemic lupus erythematosus | - | - | Y | - | - |
| Contra-indications | Contraindicated in liver dysfunction | - | - | - | - | Y |
|  | Do not give within 24hours of oral typhoid vaccines | - | - | Y | Y | - |
|  | Contraindicated during pregnancy | Y | Y | Y | Y | Y |
|  | Contraindicated in children < 8 years | Y | Y | Y^g^ | Y | Y |
|  | Contraindicated during breast-feeding | Y | Y | Y | - | - |
| MEFLOQUINE | | | | | | |
| Effectiveness | Use mefloquine in areas with chloroquine-resistant malaria | Y | Y | - | Y | Y |
|  | Mefloquine is 90% or more effective against malaria | Y | - | Y | - | - |
| Administration | Take once weekly | Y | - | Y | Y | Y |
|  | Take 1 week before entering malarial region, during period of exposure, and for 4 weeks after leaving malarial region | Y | Y | Y^h^ | Y^i^ | Y |
|  | Consider initiating mefloquine 3 weeks before departure to determine side effects | Y | Y | Y | Y^j^ | Y |
| Safety | Safe in pregnancy but caution in first trimester | Y | Y | Y | Y | Y |
|  | May be given to children over 5kg | Y | Y | Y | Y^k^ | Y |
|  | Safe in breastfeeding | - | - | - | - | Y |
|  | Commonly no side effects or only mild and temporary ones | Y | Y | - | - | - |
|  | Minor side effects include nausea, strange vivid dreams, dizziness, mood changes, insomnia, headache and diarrhoea | Y | Y | - | Y | - |
|  | Rarely causes seizure, psychosis and suicidal ideation | Y | Y | - | Y | Y |
|  | Caution with anticoagulants, as may potentiate the effect | Y | - | - | - | - |
|  | Caution with chloroquine, quinine-like drugs, ketoconazole, HIV protease inhibitors; some antiepileptics, zyban | Y | - | Y | Y | Y |
|  | Caution in occupations requiring fine coordination, flying an aircraft, or in scuba diving | Y | - | Y | - | - |
| Contra-indications | Contraindicated in individuals with a history of psychiatric disorder or seizure disorder | Y | Y | Y | Y | Y |
|  | Contraindicated in those with cardiac conduction delays or arrhythmia | Y | - | - | Y | - |
|  | Do not give with live vaccines | Y | - | - | - | Y |
| STAND-BY EMERGENCY TREATMENT (SBET) OR SELF-TREATMENT | | | | | | |
| Eligible travellers | Travellers who would have difficulty accessing medical services within 24hrs | Y | Y | Y | Y | Y |
|  | Other suggested eligible include: frequent short visits over prolonged period of time; suboptimal malaria chemoprophylaxis; low risk of infection | N | Y | N | N | Y |
| Advice | Clear directions to be given to traveller on symptoms, administration, side effects | Y | Y | Y | N | Y |
| Medical advice | Seek medical advice if get fever within 24 hours | Y | Y | Y | N | Y |

*Y=recommended in guidelines; N=alternative recommended; - not discussed

1. Discusses P. vivax only
2. PHE recommends against the use of chloroquine-proguanil
3. WHO states start 1 day before entering region
4. Safety profile over 9 months unknown
5. 8% to 15% experience gastrointestinal symptoms
6. USA states not recommended in <5kg
7. UK guidance states over 12 years
8. 3 weeks before
9. ≥2 weeks before
10. ≥2 weeks before
11. Can be used in children of all ages
