# Supplementary material for: Preventing malaria in international travellers: an evaluation of published English-language guidelines
Source: BMC Public Health. 2014 Nov 3;14:1129. doi: 10.1186/1471-2458-14-1129 (PMC4226897; doi:10.1186/1471-2458-14-1129)
Supplement: Supplementary file 2 — Additional file 2: Consensus between guidelines for non-drug interventions. (DOC 115 KB) [file 12889_2014_7210_MOESM2_ESM.doc]

***Additional file*** 2: Consensus between guidelines for non-drug interventions

| Non-drug Interventions | Advice | Does guideline make recommendation? | | | | |
| --- | --- | --- | --- | --- | --- | --- |
|  |  | CA | HK | UK | USA | WHO |
| GENERAL GUIDANCE | | | | | | |
| Prophylaxis | Take travel history give advice accordingly | Y | Y | Y | Y | Y |
|  | Incorporate traveller’s medical history into risk assessment | Y | Y | - | - | - |
|  | Initiate prophylaxis in advance if concerns about tolerability | Y | - | - | - | - |
| Traveller education | Make travellers aware of risk of acquiring malaria (A) | Y | - | Y | - | Y |
|  | Importance of preventing being bitten (B) | Y | - | Y | - | Y |
|  | Importance of taking chemoprophylaxis, as appropriate (C) | Y | - | Y | - | Y |
|  | Importance of prompt diagnosis and treatment if ill (D) | Y | Y | Y | - | Y |
|  | Provide additional education to those least likely to be compliant with chemoprophylaxis (backpackers, immigrants visiting country of origin, long-term travellers, <40 years old) | Y | - | - | - | - |
|  | Use visual aids and written information when educating | - | - | Y | - | - |
|  | Discuss choices of chemoprophylaxis | - | Y | Y | - | - |
| Pregnancy | Pregnant women should seek individual medical advice | - | - | - | - | Y |
|  | Advise pregnant women to avoid travel to malarial areas | Y | Y | Y | Y | Y |
|  | Advise pregnant women about increased susceptibility to mosquito bites and need for additional prevention interventions | - | - | Y | - | Y |
| Children | People travelling with children should seek individual medical advice | - | - | - | - | Y |
|  | Advise young children to avoid travel to malarial areas | Y | Y | Y | - | Y |
|  | Advise use of personal protective measures for all children who travel to malaria-endemic areas | Y | Y | Y | Y | Y |
|  | Advise that all children should receive chemoprophylaxis | Y | Y | Y | Y | Y |
|  | Advise about specific administration of tablets for children | Y | - | - | Y | Y |
| Insecticide-treated bed nets (ITNs) | | | | | | |
| Effectiveness | Sleep under bed nets | Y | Y | Y | Y | Y |
|  | Use insecticide-treated bed nets | Y | Y | Y | Y | Y |
| Pregnancy | Pregnant woman should sleep under bed nets | Y | - | - | - | Y |
| Children | Children should sleep under bed nets, including prams & cots | Y | - | - | Y | Y |
| Implement-ation | Ensure net is intact, with no holes | Y | - | Y | - | Y |
|  | Ensure net is tucked into mattress | Y | - | Y | Y | Y |
|  | ITNs will need retreating with insecticide, dependent on the product | Y | - | Y | Y | - |
| Insecticide-treated clothing | | | | | | |
| Effectiveness | Use insecticide-treated clothing is advised | Y | Y | Y | Y | Y |
| Safety | Follow instructions for treating fabrics to avoid damage | - | - | - | - | Y |
| Implement-ation | Treat clothing 24–48 hours in advance of travel | - | - | - | Y | - |
|  | Application of repellent to clothing lasts longer than applied to skin | - | - | N^a^ | - | Y |
| Skin-applied chemical repellents e.g. DEET | | | | | | |
| Effectiveness | Use DEET as the first choice repellent | Y | Y | Y | Y | Y |
|  | Use Picaridin or Icaridin as an alternative | Y | Y | Y | Y | Y |
|  | Use IR3535 as an alternative to DEET | - | - | Y | Y | Y |
|  | Use lemon eucalyptus oil as an alternative to DEET | Y | Y | Y | Y | - |
|  | Use soybean oil 2% repellents as an alternative to DEET | Y | Y | - | - | - |
|  | Do not use repellents containing citronella oil | Y | - | Y | - | - |
| Safety | Apply DEET according to product directions to minimise adverse effects | Y | - | Y | - | - |
|  | Do not ingest/inhale or allow repellents to come into contact with eyes, mouth, wounds or deep skin folds | - | - | Y | Y | Y |
|  | Beware that DEET can damage some plastic and clothing | - | - | Y | - | - |
| Pregnancy | Use of DEET is encouraged for all pregnant women | Y | - | Y | Y | Y |
| Children | Advise judicious use of DEET for children of any age | Y | N^b^ | N^c^ | N^c^ | Y |
|  | Use up to 30% DEET-containing products | Y | Y^d^ | N^e^ | - | - |
| Implement-ation | Use extended-duration DEET formulations | Y | - | - | - | - |
|  | Use DEET concentrations up to 35% | Y | Y | N^f^ | N^f^ | - |
|  | Reapply DEET regularly if sweating | - | - | Y | - | Y |
|  | Do not use DEET and sunscreen combination products | Y | - | - | Y | - |
|  | Apply sunscreen first, followed by DEET if both are required | Y | - | Y | Y | - |
| Aerosol insecticides | | | | | | |
| Effectiveness | Use aerosol sprays to kill mosquitoes indoors | - | Y | Y | Y | Y |
| Implement-ation | Spray rooms before dusk to kill any mosquitoes which may have entered the accommodation during the day | - | - | Y | Y | Y |
| Air conditioning | | | | | | |
| Effectiveness | Sleep in air conditioned rooms to reduce risk of mosquito bite | - | - | Y | Y | Y^g^ |
| Mosquito coils and vapoUrising mats | | | | | | |
| Effectiveness | Burn mosquito coils to kill mosquitoes indoors | - | Y | Y | - | Y |
|  | Use vaporising mats instead of coils, if electricity available | - | - | Y | - | Y |
| Other interventions | | | | | | |
| Sleep in accommodation with intact screens on windows | | Y | Y | Y | Y | Y |
| Wear protective clothing (long shirts/trousers) to prevent mosquito bites | | Y | Y | Y | Y | Y |
| Wear light coloured clothing to prevent mosquito bites | | Y | Y | N^h^ | - | - |
| Do not use electronic buzzers and electrocuting devices to prevent mosquito bites | | Y | - | Y | - | - |
| Do not use impregnated wrist/ankle bands to prevent mosquito bites | | Y | - | - | - | - |
| Do not use odour-baited mosquito traps to prevent mosquito bites | | Y | - | - | - | - |
| Do not use herbal remedies to prevent mosquito bites to prevent mosquito bites | | Y^i^ | - | Y | - | - |
| Do not use orally administered vitamin B1 to prevent mosquito bites | | Y | - | Y | - | - |
| Do not use skin moisturizers that do not contain an approved active repellent to prevent mosquito bites | | Y | - | - | - | - |
| Do not use garlic to prevent mosquito bites | | - | - | Y | - | - |
| Do not use tea tree oil to prevent mosquito bites | | - | - | Y | - | - |

* Y=guidelines recommend this; N=guidelines recommend alternative; - guideline does not mention

a. UK guidelines states opposite, cotton clothing can be sprayed with DEET but its duration on clothing is shortened due to its volatility

b. HK guidelines state that DEET may be associated with neurotoxicity in children

c. UK &USA guidelines advises DEET not recommended for infants below the age of 2 months

d. >12yrs use 30%, <12yrs use 10%

e. UK guidelines advises use of 50% in children over 2months

f. UK and USA guidelines advises use of up to 50% DEET

g. WHO states in air-conditioned hotels, other precautions are not necessary indoors

h. UK guidance states no evidence for this

i. Specifically geranium plant
